# Population genetics of *Glossina palpalis palpalis* in sleeping sickness foci of Côte d’Ivoire before and after vector control

**DOI:** 10.1016/j.meegid.2019.103963

**Published:** 2019-11

**Authors:** Djakaridja Berté, Thierry De Meeûs, Dramane Kaba, Modou Séré, Vincent Djohan, Fabrice Courtin, Martial N'Djetchi Kassi, Mathurin Koffi, Vincent Jamonneau, Bi Tra Dieudonné Ta, Philippe Solano, Eliezer Kouakou N'Goran, Sophie Ravel

**Affiliations:** a Institut Pierre Richet/Institut National de Santé Publique, Bouaké, Côte d’Ivoire; b Université Felix Houphouët-Boigny, Abidjan, Côte d’Ivoire; c Intertryp, IRD, Cirad, Univ Montpellier, Montpellier, France; d Université de Dédougou (UDDG), Dédougou, Burkina Faso; e Laboratoire des Interactions Hôte-Microorganisme-Environnement et Evolution, Unité de Formation et de Recherche Environnement, Université Jean Lorougnon Guédé, Daloa, Côte d’Ivoire

**Keywords:** Tsetse flies, Population genetics, Control, Dispersal, Resistance, Côte d'Ivoire

## Abstract

*Glossina palpalis palpalis* remains the major vector of sleeping sickness in Côte d'Ivoire. The disease is still active at low endemic levels in Bonon and Sinfra foci in the western-central part of the country. In this study, we investigated the impact of a control campaign on *G. p. palpalis* population structure in Bonon and Sinfra foci in order to adapt control strategies. Genetic variation at microsatellite loci was used to examine the population structure of different *G. p. palpalis* cohorts before and after control campaigns. Isolation by distance was observed in our sampling sites. Before control, effective population size was high (239 individuals) with dispersal at rather short distance (731 m per generation). We found some evidence that some of the flies captured after treatment come from surrounding sites, which increased the genetic variance. One Locus, GPCAG, displayed a 1000% increase of subdivision measure after control while other loci only exhibited a substantial increase in variance of subdivision. Our data suggested a possible trap avoidance behaviour in *G. p. palpalis*. It is important to take into account and better understand the possible reinvasion from neighboring sites and trap avoidance for the sake of sustainability of control campaigns effects.

## Highlights

• In Côte d’Ivoire HAT foci, *Glossina palpalis palpalis* show relatively important effective population size and modest dispersal before control.
• Genetic data analyses show that flies captured after control partly (at least) come from surrounding sites.
• One allele at Locus GPCAG displayed a 328% allele frequency increase after control.
• This locus (GPCAG) seems associated to resistance to control, by a mechanism which remains to be identified.

## Graphical abstract

**Figure:**
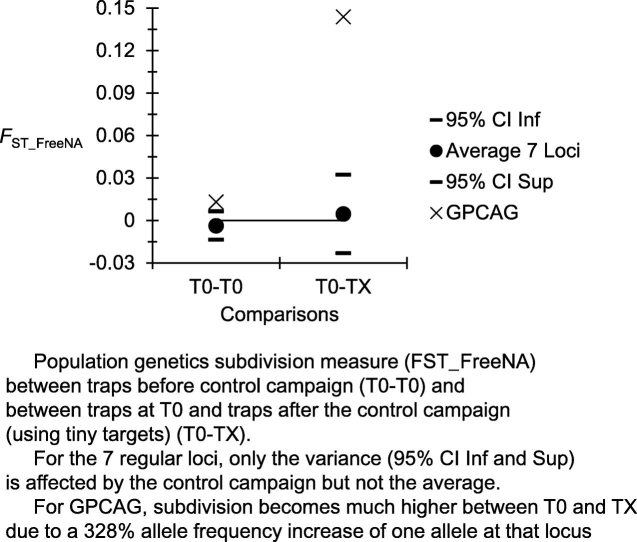

## 1 Introduction

Human African Trypanosomiasis (HAT) is a centuries-old disease that has affected the lifestyle of people in sub-Saharan Africa (Steverding, 2008). It is a parasitic disease due to two subspecies of *Trypanosoma brucei* transmitted by tsetse flies belonging to the *Glossina* genus. There are two forms of HAT: one, known as gambiense HAT, due to *T. brucei gambiense*, is endemic in West and Central Africa and causes over 95% of current cases; the other, known as rhodesiense HAT, due to *T. brucei rhodesiense*, is endemic in East and Southern Africa and accounts for the remainder of cases (Büscher et al., 2017).

The disease reemerged at the end of the 1990s, but renewed efforts from endemic countries brought the disease under control again (Franco et al., 2018). In this context, sustainable elimination of the gambiense HAT was considered as a feasible target for 2030 (Franco et al., 2014).

Tsetse control has recently become a key component of the overall activities of HAT control (Solano et al., 2013; Bouyer et al., 2015; Courtin et al., 2015). However, many tsetse control efforts were not sustainable due to either flies surviving the initial interventions, or flies immigrating from untreated regions, or both (Hargrove, 2003; Adam et al., 2014; Meeûs et al., 2019), except when control itself is sustained (Simo and Rayaisse, 2015; Meyer et al., 2016). This lead to the fact that in 2015, animal trypanosomiasis was still an important issue in West and Central Africa (Simo and Rayaisse, 2015). The knowledge of the genetic structure of a target population can facilitate decision-making (Mccoy, 2008; Solano et al., 2010a; Solano et al., 2010b).Quantifying exchanges of individuals among subpopulations gives information on the isolation status and structure of the studied population. These population parameters are important for medical entomologists as they may have consequences on the epidemiology and control of vector borne diseases (De Meeûs et al., 2007; Solano et al., 2010b; Chevillon et al., 2012; Krafsur and Maudlin, 2018; De Meeûs et al., 2019; Manangwa et al., 2019).

In Côte d'Ivoire, the development of cash crops (coffee and cocoa) in western part of the country has led to profound changes in the biotopes that are favorable to tsetse flies (Courtin et al., 2008; Cecchi et al., 2009) so that the country is the second most affected by HAT in West Africa (Simarro et al., 2010). Bonon and Sinfra foci are still active at low endemic level, since 11 cases have been reported in both foci in the last five years according to National Program for HAT Elimination. In order to accelerate elimination, in addition to active and passive HAT cases screening, a tsetse control campaign based on the use of impregnated tiny targets (Esterhuizen et al., 2011; Rayaisse et al., 2011; Rayaisse et al., 2012) has been firstly set in the Bonon focus in February 2016, then in Sinfra in May 2017.

In this study, we aimed to assess how vector control can affect the population genetic structure of *Glossina palpalis palpalis* in the Bonon focus. Sinfra subsamples were added to allow widening the studied area and get more precision on the initial structure of tsetse populations before any control in the Marahoue region.

## 2 Material and methods

### 2.1 Study site

**Fig. 1:**
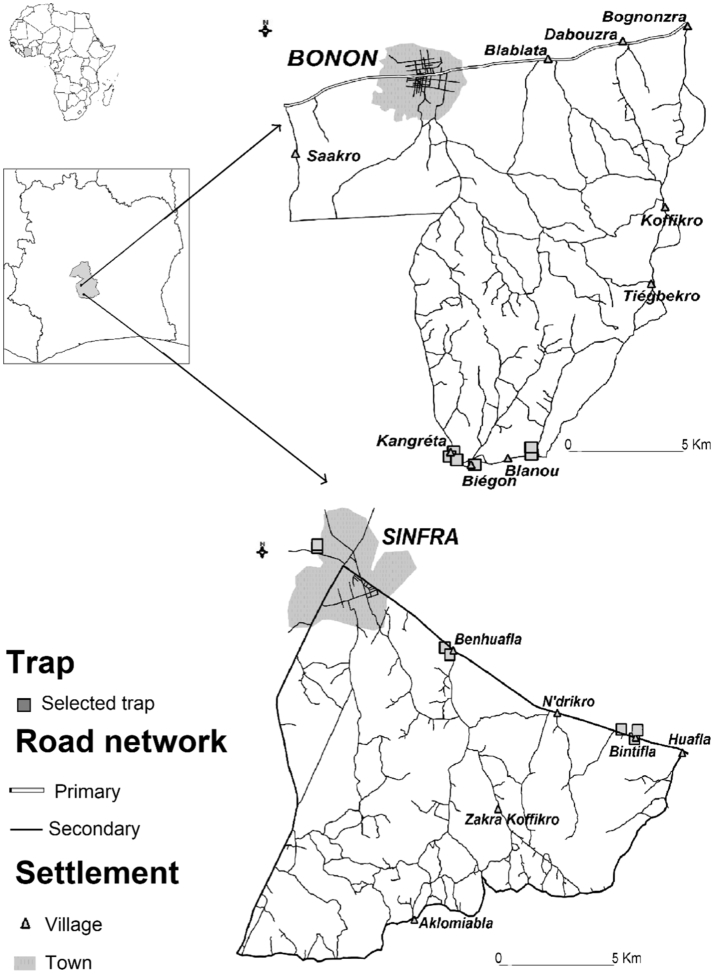
Location map of study area with selected traps.

The study was carried out in the Bonon and Sinfra HAT foci located in the western-central part of Côte d'Ivoire (Fig. 1). These foci belong to the Marahoué region, which is located in a mesophilic forest area, although forest has now almost disappeared, replaced by coffee and cocoa plantations. In these foci and surroundings, many livestock farms raising pigs, goats, sheep and cattle are present (Courtin et al., 2005; N'Djetchi et al., 2017). Due to the cash crops interests (coffee, cocoa, bananas, etc.) and associated human settlements, favoring tsetse-human contact, HAT found ideal geographic conditions for its development, and Bonon and Sinfra became the most active foci in Côte d'Ivoire at the beginning of the 1990s (Dje et al., 2002; Solano et al., 2003; Kaba et al., 2006). Thanks to control efforts, only few cases are now reported each year in these foci (Koffi et al., 2016).

### 2.2 Sampling

Sampling extended from May 2015 to March 2017. If we consider a 2 months length for a tsetse generation (Williams, 1990; Krafsur, 2009), our sampling contain different tsetse fly cohorts: cohorts 1, 7, 8, 9, 10 and 11. In Bonon focus, the first entomological survey was carried out in June 2015 (T0, cohort 1), before the control campaign. Tsetse control campaign took place in February 2016 and then 30 sentinel traps were deployed at chosen sites in order to monitor the evolution of tsetse flies densities, among which 25 were used for the present study. Evaluations were done every three months allowing us to define T1, T2, T3 and T4. T1 (June 2016, cohort 7) corresponded to the first survey after control, T2 (September 2016, cohort 8) to the second survey, T3 (December 2016, cohort 10) to the third survey and T4 (March 2017, cohort 11) to the fourth entomological survey after control. In Sinfra, two entomological surveys were done before tsetse control. The first was done in May 2015 (T0, cohort 1) and the second in November–December 2016 (T0_bis, cohort 9). Sinfra subsamples allowed getting more precision on the initial structure of tsetse populations of the Marahoue region before control. Tsetse flies were sampled using Vavoua traps deployed during two consecutive days in tsetse fly favorable biotopes. In total, 17 and 8 traps were used in Bonon and Sinfra respectively (Table 1). For the population genetics analyses, we used lies captured in 14 traps: 6 in Bonon at T0 and 8 in Sinfra. In Bonon, we used captured flies from 4 traps at T1, 5 traps at T2, 3 traps at T3, and 3 traps at T4. These 30 subsamples varied in size from 1 to 25 flies (13 on average), but most (21) contained >9 flies. Surface of sampling was computed for each site (Table 1) with the longer distance between two traps (*D*_max_) taken as the radius of the disc that contained all traps of the site (*S*_*S*_ = *π* × *D*_max_^2^). Census densities (*D*_*c*_) were computed as the number of captured flies per site (*N*_*c*_) divided by *S*_*S*_ (Table 1). The comparison between densities at T0 and the others (T1-T4) was undertaken with a Wilcoxon rank sum test with continuity correction with R-Commander package (Fox, 2005; Fox, 2007) for R (R-Core-Team, 2018).

**Table 1:** Number of *Glossina palpalis palpalis* sampled in each site -*N*_*c*_, description of sampling, surface of sampling (*S*_S_) (in km^2^), observed densities (*D*_*c*_) and number of genotyped flies (*N*_*G*_). Treatment status (T) are indicated and GPS coordinates (Long, Lat) are given in decimal degrees.

Focus	T	Site	Trap	Long	Lat	*S*_S_	*N*_*c*_	*D*_*c*_	*N*_*G*_
Bonon	T0	Biegon	BE4017	−6.0298	6.7966	2.9326	9	85	0
Bonon	T0	Biegon	BE4019	−6.0377	6.7930	2.9326	38	85	0
Bonon	T0	Biegon	BG4018	−6.0344	6.7962	2.9326	203	85	22
Bonon	T0	Blanou	BE4015	−6.0250	6.7933	1.3513	38	33	0
Bonon	T0	Blanou	BG4013	−6.0239	6.7979	1.3513	0	33	0
Bonon	T0	Blanou	BG4014	−6.0231	6.7989	1.3513	5	33	0
Bonon	T0	Blanou	BG4016	−6.0249	6.7975	1.3513	1	33	0
Bonon	T0	Dianou	BE4008	−6.0170	6.7996	2.1569	259	336	15
Bonon	T0	Dianou	BE4012	−6.0124	6.7962	2.1569	63	336	0
Bonon	T0	Dianou	BG4005	−6.0149	6.7989	2.1569	0	336	0
Bonon	T0	Dianou	BG4006	−6.0157	6.7997	2.1569	7	336	0
Bonon	T0	Dianou	BG4007	−6.0170	6.8007	2.1569	79	336	0
Bonon	T0	Dianou	BG4009	−6.0164	6.8018	2.1569	111	336	0
Bonon	T0	Dianou	BG4010	−6.0171	6.8020	2.1569	206	336	14
Bonon	T0	Kangreta	BE4021	−6.0422	6.7989	0.3256	53	1056	16
Bonon	T0	Kangreta	BE4022	−6.0399	6.7978	0.3256	190	1056	21
Bonon	T0	Kangreta	BG4020	−6.0409	6.8006	0.3256	101	1056	15
Bonon	T1	Biegon	BG4018	−6.0344	6.7962	2.9326	22	8	18
Bonon	T1	Dianou	BE4008	−6.0170	6.7996	2.1569	1	1	1
Bonon	T1	Dianou	BG4010	−6.0171	6.8020	2.1569	2	1	2
Bonon	T1	Kangreta	BE4021	−6.0422	6.7989	0.3256	1	126	0
Bonon	T1	Kangreta	BE4022	−6.0399	6.7978	0.3256	3	126	3
Bonon	T1	Kangreta	BG4020	−6.0409	6.8006	0.3256	37	126	7
Bonon	T2	Biegon	BG4018	−6.0344	6.7962	2.9326	20	7	18
Bonon	T2	Dianou	BE4008	−6.0170	6.7996	2.1569	7	6	5
Bonon	T2	Dianou	BG4010	−6.0171	6.8020	2.1569	6	6	6
Bonon	T2	Kangreta	BE4021	−6.0422	6.7989	0.3256	14	86	11
Bonon	T2	Kangreta	BE4022	−6.0399	6.7978	0.3256	1	86	0
Bonon	T2	Kangreta	BG4020	−6.0409	6.8006	0.3256	13	86	11
Bonon	T3	Biegon	BG4018	−6.0344	6.7962	2.9326	0	0	0
Bonon	T3	Dianou	BE4008	−6.0170	6.7996	2.1569	10	21	6
Bonon	T3	Dianou	BG4010	−6.0171	6.8020	2.1569	36	21	17
Bonon	T3	Kangreta	BE4021	−6.0422	6.7989	0.3256	39	141	24
Bonon	T3	Kangreta	BE4022	−6.0399	6.7978	0.3256	1	141	1
Bonon	T3	Kangreta	BG4020	−6.0409	6.8006	0.3256	6	141	6
Bonon	T4	Biegon	BG4018	−6.0344	6.7962	2.9326	8	3	7
Bonon	T4	Dianou	BE4008	−6.0170	6.7996	2.1569	17	9	15
Bonon	T4	Dianou	BG4010	−6.0171	6.8020	2.1569	2	9	2
Bonon	T4	Kangreta	BE4021	−6.0422	6.7989	0.3256	0	3	0
Bonon	T4	Kangreta	BE4022	−6.0399	6.7978	0.3256	0	3	0
Bonon	T4	Kangreta	BG4020	−6.0409	6.8006	0.3256	1	3	0
Sinfra	T0	Benhuafla	SE2002	−5.8812	6.5960	0.2813	154	874	22
Sinfra	T0	Benhuafla	SE2004	−5.8796	6.5938	0.2813	92	874	19
Sinfra	T0	Bintifla	SE1012	−5.8189	6.5685	1.1297	55	283	19
Sinfra	T0	Bintifla	SE1013	−5.8178	6.5710	1.1297	207	283	25
Sinfra	T0	Bintifla	S1023	−5.8232	6.5712	1.1297	20	283	16
Sinfra	T0	Bintifla	S1031	−5.8178	6.5710	1.1297	38	283	14
Sinfra	T0	Ville	S5030	−5.9232	6.6272	0.0351	27	2279	13
Sinfra	T0	Ville	S5031	−5.9233	6.6263	0.0351	53	2279	12

Three legs were removed from each fly and stored in 70% ethanol tubes labelled with a code containing the trap number followed by individual fly number and the sampling date. In total 403 tsetse individuals were analyzed (Table 1 and Supplementary Table S1). All flies were identified as *Glossina palpalis palpalis* according to morphological criteria (Pollock, 1982).

### 2.3 Genotyping

Tsetse flies were genotyped at 10 microsatellite loci (Supplementary Table S1, Supplementary Table S2). Locus X55-3 is from (Solano et al., 1997). Loci XpGp13 and pGp24 come from (Luna et al., 2001) and GPCAG from (Baker and Krafsur, 2001). Loci, B3, XB104, XB110 and C102 were kindly supplied by A. Robinson, Insect Pest Control Laboratory (formerly Entomology Unit), Food and Agricultural Organization of the United Nations/International Atomic Energy Agency [FAO/IAEA], Agriculture and Biotechnology Laboratories, Seibersdorf, Austria. Finally, loci pGp20 and pGp27 came from a microsatellite bank of *G. palpalis gambiensis* (S. Ravel, personal communication). Those the name of which begins with the letter X are X-linked. We thus analyzed two data sets: one without these loci but with all individuals and the other with females only and all loci. More characteristics for describing the loci used can be found in the Supplementary Table S2.

In the laboratory, legs were dried and then subjected to chelex treatment as previously described (Ravel et al., 2007). The 10 PCR reactions were then carried out in a thermocycler (MJ Research, Cambridge, UK) in 20 μl final volume, using 10 μl of the diluted supernatant from the extraction step as template. After PCR amplification at the microsatellite loci, allele bands were routinely resolved on ABI 3500 XL sequencer (Applied Biosystems, USA). This method allows multiplexing and the use of four dyes (blue, red, green and yellow). Allele calling was done using GeneMapper V 4.1software (Applied Biosystems) and the size standard GS600LIZ short run.

In order to confirm subspecies determination, we amplified partial sequences of ITS1 following the protocol described by Dyer et al. (2008).

All data and genotypes are available in the Supplementary Table S1. In total, 403 flies, including 309 females and 94 males, were genotyped.

### 2.4 Data analyses

All data were formatted for Create (Coombs et al., 2008) and transformed in the appropriate format for subsequent analyses.

Significance of linkage disequilibrium (LD) between locus pairs was assessed with the *G*-based test of Fstat 2.9.4 (Goudet, 2003) an updated version of Fstat (Goudet, 1995) with 10,000 permutations. The *G*-based test allows obtaining a global test across subsamples (for each pair of loci) and is more powerful than other combining procedures (De Meeûs et al., 2009). There are as many tests as locus pairs, i.e. *L*(*L*-1)/2 (15 if the number of loci *L* = 6) These tests are not independent. We used the Benjamini and Yekutieli false discovery rate (FDR) procedure (Benjamini and Yekutieli, 2001) that is appropriate in case of non-independent test series. The corrected *p*-values were computed with R (R-Core-Team, 2018).

For a hierarchy with three levels (individuals in subsample in total sample), three *F*-statistics (Wright, 1965) can be defined: *F*_IS_, which measures inbreeding of individuals relative to inbreeding of subsamples; *F*_ST_, which measures inbreeding of subsamples relative to total inbreeding; and *F*_IT_, which measures inbreeding of individuals relative to total inbreeding. Deviations of genotypic proportions expected under local panmixia are measured by *F*_IS_, while *F*_ST_ measures the effect of subdivision (genetic isolation between subsamples) and *F*_IT_ reflects the combination of both (e.g. (De Meeûs et al., 2007)). Under the null hypothesis (panmixia and no subdivision), all these statistics are expected to be null. Otherwise *F*_IS_ and *F*_IT_ can vary from −1 (heterozygote excess) to +1 (homozygote excess) and *F*_ST_ from 0 (all subsamples share similar allele frequencies) to +1 (all subsamples fixed for one or the other allele). In any case, the three statistics are linked by the famous relationship: 1-*F*_IT_ = (1-*F*_IS_)(1-*F*_ST_) (e.g. (De Meeûs, 2018)).

In dioecious species (like tsetse flies), heterozygote excess is expected over all loci in small random-mating subpopulations (e.g. (De Meeûs et al., 2007)). Multilocus positive *F*_IS_ (homozygote excess) can be produced by systematic mating between related individuals like sib-mating (e.g. (De Meeûs et al., 2007)). It can also come from the admixture, in each subsample, of individuals that belong to genetically divergent entities (subpopulations, subspecies or species) (Wahlund effect) (e.g. (De Meeûs et al., 2007; De Meeûs, 2018)).

Technical problems, like null alleles, stuttering, short allele dominance or allele dropouts unevenly affects some loci, producing a positive *F*_IS_ with an important variation across loci (De Meeûs, 2018).

A positive value for *F*_ST_ suggests that the total population is subdivided, for instance, into *n* subpopulations of effective size *N*_e_ and an immigration rate of *m*. In an Island model of migration (with no spatial structure) (Wright, 1951) at mutation-drift equilibrium, we expect *F*_ST_ = 1/[4*N*_*e*_(*m* + *u*) + 1], where *u* is the mutation rate of the locus (e.g. (De Meeûs et al., 2007)). If *u* < <*m*, the number of immigrants *N*_*e*_*m* can be extracted as *N*_*e*_*m* = (1 − *F*_ST_)/(4*F*_ST_).

Wright's *F*-statistics were estimated through Weir and Cockerham's unbiased estimators (Weir and Cockerham, 1984). It is worthy of note that *θ*, *F*_ST_ estimator, can display negative values. This happens when subsamples share more similar genetic composition than would be expected if the different subsamples were randomly drawn from the same subpopulation, i.e. when alleles are more related between than within subsamples ((Weir, 1996) page 175).

For *F*-statistics, significant departure from 0 was tested by randomizing alleles among individuals within subsample (deviation from local random mating test) or of individuals among subsamples within the total sample (population subdivision test) (10,000 permutations in each case). The *p*-value then corresponded to the number of times a statistic measured in randomized samples was as big as or bigger than the observed one (unilateral tests). For *F*_IS_, the statistic used was *f* (Weir and Cockerham's *F*_IS_ estimator). To test for subdivision, we used the *G*-based test (Goudet et al., 1996) over all loci, which is the most powerful procedure when combining tests across loci (De Meeûs et al., 2009).

To compute 95% confidence intervals (95%CI) of *F*-statistics, we used the standard error of *F*_IS_ (StrdErrFIS) and *F*_ST_ (StrdErrFST) computed by jackknife over populations, and 5000 bootstraps over loci as described elsewhere (De Meeûs et al., 2007). Since jackknife's computation of 95%CI assumes a normal distribution of the parameters and because *F*-statistics do not follow such a distribution, these confidence intervals were only used to graphically visualize parameter variation across subsamples and not for statistical comparisons. Bootstrap's 95%CI does not require that the data follow any distribution and is thus statistically valid.

In case of significant homozygote excess and linkage disequilibrium we have tried to discriminate demographic from technical causes with the determination key proposed by De Meeûs (2018). In case of null alleles, both *F*_IS_ and *F*_ST_ are augmented, StrdErrFIS is at least twice StrdErrFST (jackknives over loci)_,_ a positive correlation is expected between *F*_IS_ and *F*_ST_ as is expected a positive correlation between *F*_IS_ and the number of missing data (putative null homozygotes). If such correlations do not exist and if StrdErr*F*_IS_ > StrdErr*F*_ST_, then a Wahlund effect better explains the data. The significance of correlations was tested with a unilateral (*ρ* > 0) Spearman's rank correlation test with R. The presence of null alleles was also looked for with MicroChecker v 2.2.3 (Van Oosterhout et al., 2004) and null allele frequencies estimated with Brookfield's second method (Brookfield, 1996). The adjustment between observed and expected numbers of missing data was tested with a unilateral exact binomial test under R with the alternative hypothesis: “there are not enough missing data as expected if heterozygote deficits were entirely explained by null alleles under panmixia”. MicroChecker also checks for stuttering and short allele dominance (SAD). Short allele dominance was also assessed with unilateral (*ρ* < 0) Spearman's rank correlation test between allele size and *F*_IT_, which is more powerful than other alternatives (Manangwa et al., 2019). In case of SAD, a negative correlation is expected (unilateral tests).

For local population structure studies, we used all subsamples, while for population subdivision studies we kept only comparisons between subsamples from the same cohort.

When null alleles are present, unbiased estimation of subdivision and/or of isolation by distance were obtained with the ENA correction for *F*_ST_ estimates and the INA correction for Cavalli-Sforza and Edwards' chord distance (Cavalli-Sforza and Edwards, 1967) *D*_CSE_ computed with FreeNA (Chapuis and Estoup, 2007). For this, all missing genotypes were converted into homozygous individuals for allele 999 as recommended (Chapuis and Estoup, 2007).

Isolation by distance was undertaken with Rousset's approach (Rousset, 1997) where, in a two dimension framework Rousset's Index *F*_ST_R_ = *F*_ST_/(1-*F*_ST_) follows *F*_ST_R_ = *a* + *b* × Ln(*D*_G_) where *a* is a constant (intercept), Ln(*D*_G_) is the natural logarithm of the geographic distance between two sites and *b* is the slope of the regression. Rousset also demonstrated that the product of the effective population density *D*_*e*_ by the average of the squared axial distance between reproducing adults and their parents $\bar{\sigma^{2}}$: *D*_*e*_×$\bar{\sigma^{2}}=$1/(4*πb*); that the neighborhood size is *Nb* = 1/*b*; and that the number of immigrants from neighboring sites at each generation is *N*_*e*_*m* = 1/(2*πb*), where *N*_*e*_ is the effective subpopulation size and *m* is the immigration rate. The parameter *σ* represents half the average parent offspring axial distance. A proxy for dispersal distances per generation *δ* can be obtained if *N*_*e*_ and average surface of a subpopulation (*S*) are known: *D*_*e*_ = *N*_*e*_/*S* and $\delta \approx 2\times \sqrt{1/\left(4\pi {\mathrm{bD}}_{e}\right)}$. For *S*, we took either the surface computed above (*S*_S_). Another possibility is to use the threshold distance (*D*_T_) between two sites for *F*_ST_ to become positive, hence *D*_T_ = *e*^-*a*/*b*^. This new parameter was then considered as the distance between the center of two neighboring subpopulations and hence to their diameter. The corresponding surface was then *S*_T_ = *π* × (*D*_T_/2)^2^.

Significance of isolation by distance was assessed through the 5000 bootstraps confidence intervals from FreeNA and with a Mantel test between *D*_CSE_ and Ln(*D*_G_) or *F*_ST_R_, computed with the INA or ENA correction by FreeNA, as recommended for microsatellite markers (Séré et al., 2017). Because some subsamples are not contemporaneous and because we kept only contemporaneous pairs, the final matrices were not squared and were thus analyzed with the menu “Mantelize it” of Fstat. Since Fstat handles bilateral tests, we converted the resulting *p*-value into a unilateral one (correlation between geographic and genetic distance is positive) by halving the bilateral *p*-value in case of positive correlation.

Effective population sizes (*N*_*e*_) were estimated through several methods and softwares. The heterozygote excess method (Balloux, 2004) could not be used due to the excessive presence of null alleles in almost all loci (see below). The Linkage disequilibrium method (Waples and Do, 2010; Peel et al., 2013) and the coancestry method (Nomura, 2008) were undertaken with NeEstimator v 2.1 (Do et al., 2014). For LD method, several threshold values are proposed for minimal allele frequencies to be used (above 0, 0.01, 0.02 and 0.05). We used the average across usable values obtained with all methods. We also used the inter and intra correlation method (Vitalis and Couvet, 2001b) with the software Estim (Vitalis and Couvet, 2001a) (available at http://www.t-de-meeus.fr/ProgMeeusGB.html). We then computed the average, minimal and maximal (MiniMax range) effective population sizes across methods weighted by the number of usable values obtained in each case. Effective population size reflects the demography and other phenomena as reproductive strategy and/or historical perturbations and its estimation also varies across methods (Krafsur and Maudlin, 2018). We nevertheless expected a good correlation between *N*_*e*_ and the census size (*N*_*c*_) of the corresponding subpopulations (De Meeûs et al., 2019).

The surface occupied by a subpopulation was inferred with *S*_S_ and *S*_T_ described above.

Effective population densities could then be computed as *D*_*e*_ = *N*_*e*_/*S*.

The effect of control campaigns on the population genetic structure of *G. p. palpalis* was assessed first with a principal component analysis (PCA) undertaken with PCA-Gen 1.2.1 (Goudet, 1999) for which the metrics of principal axes correspond to Nei's *G*_ST_ (Nei and Chesser, 1983). Significance of axes was tested according to the broken stick criterion (Frontier, 1976) and 10,000 permutations of individuals across subsamples. We also compared *F*_ST_ (corrected for null alleles) between T0 subsample pairs and pairs between T0 and TX (i.e. sampled after control campaign at times T1, T2, T3 and T4) with a Mann-Whitney-Wilcoxon *U* test with Rcmdr.

## 3 Results

### 3.1 Fly densities

**Fig. 2:**
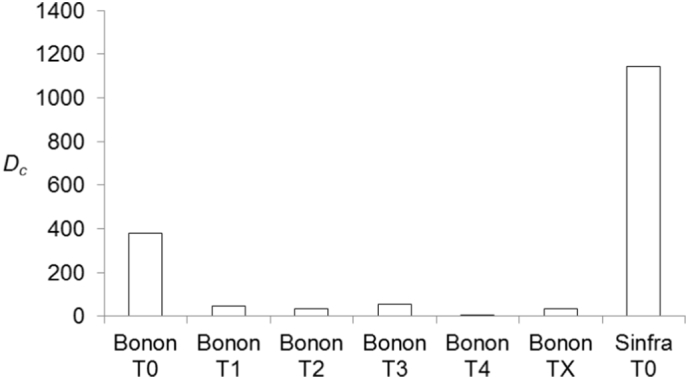
Evolution of tsetse flies apparent density per km^2^ (*D*_*c*_) from T0 (before control campaign) in Bonon and Sinfra to T4 in Bonon. In Bonon, the difference between densities at T0 and TX (X = 1–4) was significant (*p*-value = .0269) (Wilcoxon rank sum test with continuity correction).

In Bonon, fly densities decreased significantly after the control campaign (<10% of its initial value) as show in Fig. 2 (*p*-value = .0269) (see also Table 1).

### 3.2 Local population genetics with autosomal loci only in the Marahoue region

There was only one pair of loci in significant LD (*p*-value = .0406) that did not stay significant with Benjamini and Yekutieli correction (*p*-value = 1). We thus safely concluded that all markers are statistically independent.

**Fig. 3:**
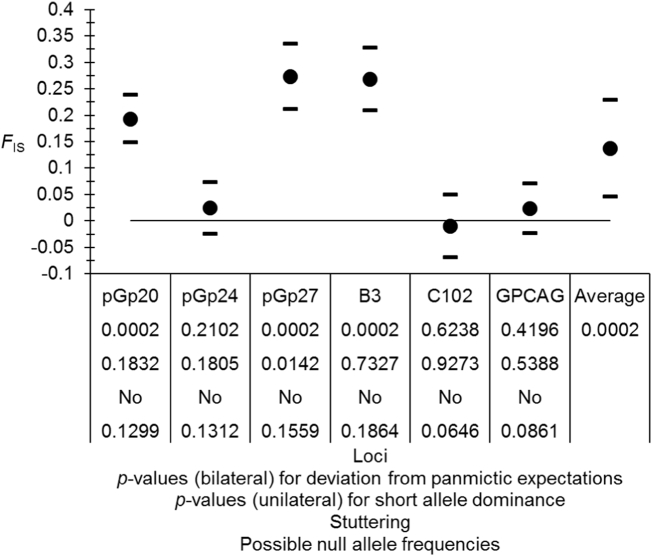
*F*_IS_ observed in subsamples of *Glossina palpalis palpalis* from Ivory Coast for autosomal loci only with jackknife's over subsamples 95% confidence intervals for each locus and 95% Bootstrap over loci confidence interval for the average. Results of panmictic tests, short allele dominance tests, stuttering detection and possible null allele frequencies are also given.

There was an important heterozygote deficit that varied considerably from one locus to the other (Fig. 3). Null alleles only explained partly these results with a StdrdErrFIS more than four times StdrdErrFST, a positive though not significant correlation between the number of observed missing genotypes and *F*_IS_ (*ρ* = 0.1429, *p*-value = .4014); and a positive though not significant correlation between *F*_IS_ and *F*_ST_ (*ρ* = 0.4058, *p*-value = .2123). No stuttering could be evidenced and a significant short allele dominance was detected for locus pGp27 (Fig. 3). This locus thus seems problematic.

### 3.3 Local population genetics with females only in the Marahoue region

There were four pairs of loci in significant LD (*p*-values <.05) that did not stay significant after Benjamini and Yekutieli correction (*p*-values >.9).

**Fig. 4:**
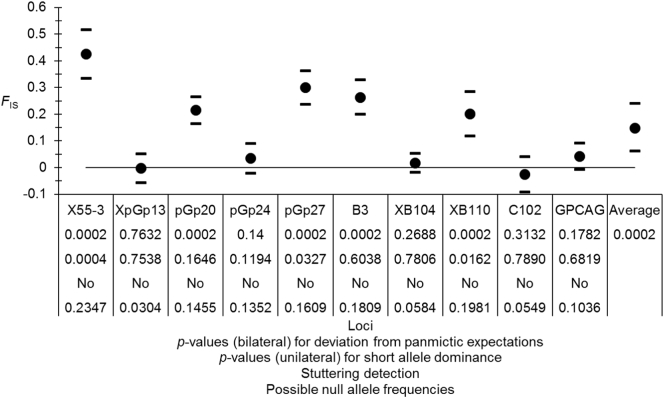
*F*_IS_ observed in subsamples of females only of *Glossina palpalis palpalis* from Ivory Coast with jackknife's over subsamples 95% confidence intervals for each locus and 95% Bootstrap over loci confidence interval for the average. Results of panmictic tests, short allele dominance tests, stuttering detection and possible null allele frequencies are also given.

The variation of *F*_IS_ was very important across the 10 loci with a global highly significant heterozygote deficit (Fig. 4). There was some evidence of the presence of null alleles with a StdrdErrFIS eight times StrdErrFST, positive correlations between the number of missing data and *F*_IS_ (*ρ* = 0.4681) and between *F*_IS_ and *F*_ST_ (*ρ* = 0.4667) though marginally not significant (*p*-value = .0862 and *p*-value = .0891 respectively). Three loci displayed short allele dominance: Loci X55–3, pGp27 and XB110 (Fig. 4). This could be expected for the first two, which displayed the highest *F*_IS_ but with too few missing genotypes. Nevertheless, for XB110, the important number of missing genotypes (the highest observed) was in agreement with null alleles as the explanation for the important *F*_IS_ observed at this locus (*F*_IS_ = 0.201 with 26 missing genotypes and null allele frequency estimated as *p*_n_ = 0.2). Furthermore, it is worth noting that the SAD signature was mainly du to the fact that all longest alleles were rare (average frequency < 0.006) and displayed a very small *F*_IT_ (average < 0.042). Considering that with a frequency of *p*_n_ = 0.2, the probability for heterozygous individuals between such alleles and a null allele is 2 × 0.2 × 0.006 = 0.0024 and the average of homozygote frequencies for these rare alleles is ~0.00005, with a total sample size of 296, this means a total expected number of falsely interpreted or true homozygous individuals for these long and rare alleles is 0.725. Thus, because null alleles affect preferentially the *F*_IS_ of the most frequent alleles, and because here these are also the shortest ones, this observation invalidates the SAD test for XB110. This locus can simply be considered as only affected by null alleles. Heterozygote deficits observed at other loci can be explained by null alleles. Subsequent subdivision measures and testing were thus done with corrections implemented by FreeNA.

**Fig. 5:**
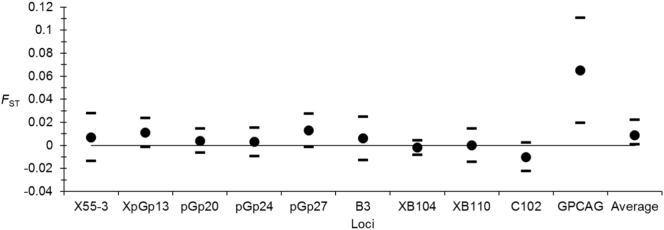
Variation of *F*_ST_ across loci for females *Glossina palpalis palpalis* from Ivory Coast.

Variations of *F*_ST_ across loci can be seen in the Fig. 5. From this figure, it can be seen that locus GPCAG's *F*_ST_ appeared completely outside the range of the nine other loci. Here, these subdivision measures are presented only for the sake of examining loci behavior. Indeed, values presented here both include temporal and spatial subdivision. Interestingly, this locus corresponds to a trinucleotide motive. It probably responds to some kind of selection.

In order to avoid possibly biased estimates, we removed loci X55–3, pGp27 and GPCAG from subsequent analyses.

To keep a number of loci above 5, we thus only studied female subsamples.

### 3.4 Population subdivision in the Marahoue region before control of female subsamples with the seven loci retained

**Fig. 6:**
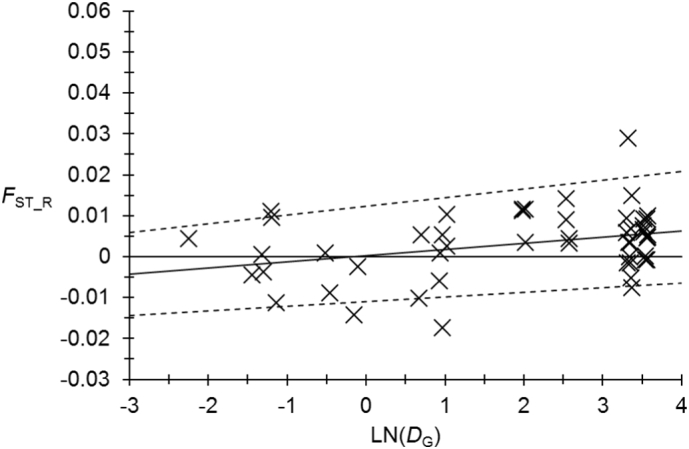
Isolation by distance for contemporaneous subsamples of *Glossina palpalis palpalis* from Bonon and Sinfra in Ivory Coast before treatment (T0). The average regression line is represented as a straight line and the 95% confidence intervals (95%CI), obtained after 5000 bootstraps over loci, are in dotted lines. Abscisses are the natural logarithms of geographic distances in km. Ordinates are Rousset's *F*_ST_R_ corrected for null alleles by FreeNA. Average slope was 0.0015 in 95%CI = [0.0011…0.0021]. Mantel test *p*-values were 0.4158 and 0.00795 for *D*_CSE_ and *F*_ST_R_ respectively.

The regression of isolation by distance is presented in the Fig. 6. The *F*_ST_R_ based tests with 95% confidence interval of the slope or with the Mantel test are significant while the *D*_CSE_ based Mantel test is not. The 95% confidence interval of the slope is a very good indicator of what occurs and *F*_ST_R_ based methods should be less powerful than with *D*_CSE_ (Séré et al., 2017), but it is highly significant here. It is thus safer to accept the alternative hypothesis of isolation by distance in this tsetse population.

Using the slope and its 95% confidence interval (*b* = 0.0015 in 95%CI = [0.0011…0.0021]), we could estimate a neighborhood size *Nb* = 667 individuals in 95%CI = [476…909] and an immigration of *N*_*e*_*m* = 106 individuals from neighboring sites (in 95%CI = [76…145]).

**Fig. 7:**
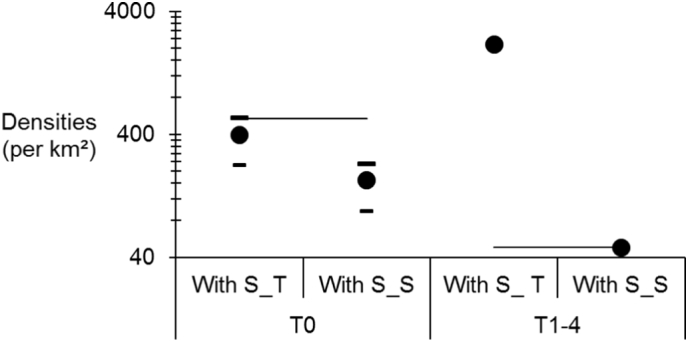
Population densities of *Glossina palpalis palpalis* with three methods: effective population density using the threshold distance for genetic subdivision (*D*_*e*_T_), effective subpopulation size using the average sampling surface of a site (*D*_*e*_S_) and census density (*D*_*c*_) (thin straight line). Effective population densities are represented with their average minimal and maximal values. Estimates are given for subsamples before the control campaign (T0) and after control (T1–4). Ordinates were scaled in log.

**Fig. 8:**
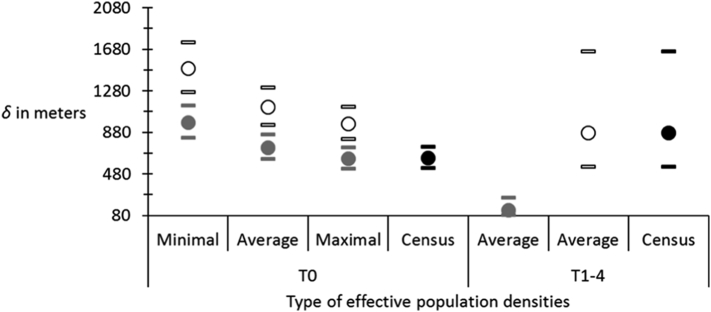
Dispersal (*δ*) of *Glossina palpalis palpalis* in Bonon and Sinfra (Ivory Coast) for different effective population densities (minimal, averaged and maximal) and different methods: using the threshold distance for genetic subdivision for defining the surface occupied by a subpopulation (grey), using the average sampling surface of the different sites (empty symbols) and using the census population density. The 95% confidence intervals computed from the isolation by distance slope after 5000 bootstraps over loci are represented as small dashes. Estimates are given for subsamples before control campaigns (T0) and after (T1–4) and were all made from Rousset's isolation by distance model between contemporaneous subsample pairs only. (For interpretation of the references to colour in this figure legend, the reader is referred to the web version of this article.)

Average effective subpopulation sizes was *N*_*e*_ = 239 ranging in MiniMax = [135…325]. Threshold geographic distance was *D*_T_ = 875 m, leading to a surface *S*_T_ = 0.602 km^2^ while the average surface of sampling in sites was *S*_S_ = 1.417 km^2^. Then effective population density *D*_*e*_S_ = 169appeared much smaller than *D*_*e*_T_ = 397, which in turn displayed values that appeared closer to the average census density *D*_*c*_ = 534 individuals per km^2^ (Fig. 7). Such inferences allowed computing dispersal as represented in the Fig. 8. Dispersal inferred from different effective population densities (Minimal, Average and Maximal) and methods (with threshold distance, sampling surface or census density) varied between 500 and 1800 m per generation. Dispersal obtained with sampling surfaces were significantly higher (1122 m on average in 95%CI [948…1310] than those computed with the threshold surface (731 m in 95%CI = [618…854]), the last being very close to values calculated with the census population density (630 m in 95%CI = [533…636]).

### 3.5 Effects of control in Bonon

**Fig. 9:**
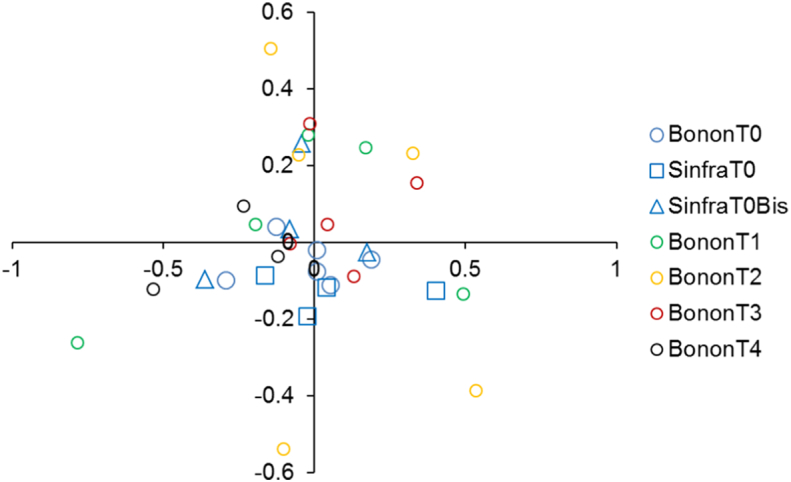
PCA Analysis result on the two first principal axes for the different subsamples before control campaigns in Bonon and Sinfra (T0, in blue) and after control campaigns (T1, T2, T3, and T4, other colors). Axis percent inertia values are 11.68 and 8.08 for axis 1 and 2 respectively. (For interpretation of the references to colour in this figure legend, the reader is referred to the web version of this article.)

The PCA graphic is represented in the Fig. 9. Although no axis was significant, it can be seen that subsamples before control campaigns occupy less space than subsamples after control campaigns, which suggests a greater genetic variance after control.

Accordingly, genetic subdivision appeared smaller between traps from T0 (*F*_ST_ = -0.005) than between traps at T0 and traps at TX (*F*_ST_ = 0.0035) but the difference was not significant (*p*-value = .1225).

Modelling isolation by distance between contemporaneous subsamples after control was uneasy because of the small number of available points. It nevertheless provided a slope the bootstrap confidence interval of which did not contain 0 (*b* = 0.0086 in 95%CI = [0.0024…0.0219]. From Fig. 7, effective population density significantly increases after treatment according to the distance threshold method (*D*_*e*_T_). On the contrary, effective population density using sampling surface (*D*_*e*_S_) significantly drops to values similar to the census density.

Regarding dispersal (Fig. 8), it significantly drops with the threshold distance method after the control campaign, while dispersal from the sampling surface method and from the census density are not significantly affected after the control campaign, with bootstrap 95%CI containing all the range of densities estimated at T0.

### 3.6 Comparison between the seven loci and GPCAG for T0/TX genetic differentiation in Bonon

**Fig. 10:**
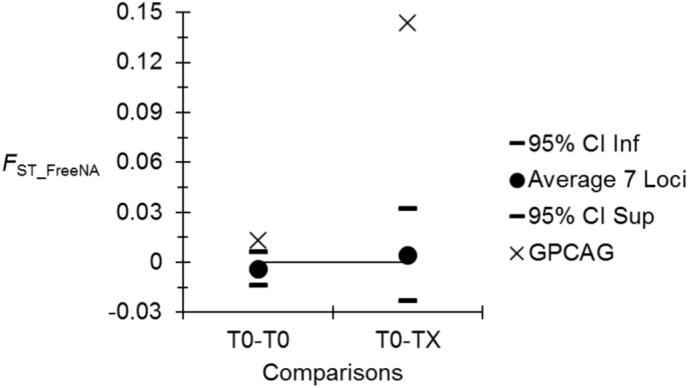
Average subdivision measures corrected for null alleles with FreeNA (*F*_ST_FreeNA_) between T0 subsample pairs (T0-T0) (before treatment campaigns) (black circles) with the seven loci kept for all analyses and 95% confidence intervals (dashes), for GPCAG alone (crosses) and between T0 and TX subsample pairs (T0-TX) (X = 1, 2, 3 or 4).

As can be seen from Fig. 10, Locus GPCAG displayed a slightly higher subdivision measure as compared to the other loci for subsamples from T0 but displayed a 1000% increase when measured between T0 and TX, while other loci displayed an unchanged average though with a much higher variance. By examining population subdivision between T1-T4 subsamples revealed absence of genetic differentiation (*F*_ST_FreeNA_ = −0.0127, *p*-value = .5843). After pooling all T0 traps together and all T1–4 together, we obtained a highly significant (*p*-value<.0001) subdivision (*F*_ST_FreeNA_ = 0.1286) for GPCAG consistent with what can be seen in Fig. 10. A glance at allele frequencies evolution reveals that this is mainly due to a 328% increase of allele 219 (from 0.146 to 0.625) at locus GPCAG after treatment.

## 4 Discussion

In the Marahoue region (Bonon and Sinfra), before control, we noted relatively high population densities and short dispersal distances as compared to other studies (De Meeûs et al., 2019). Traps selected for this study were located in villages' edge with sacred forest (i.e. religiously protected from any human activity with economic purposes) that seems to constitute highly suitable environments for tsetse flies: ideal hygrometric and shade conditions, protection from insecticide pressure existing in cultivated fields and abundant hosts as wandering pigs (Laveissière et al., 1985; Sané et al., 2000a, Sané et al., 2000b). Flies may not need to disperse much because they get all they need there and/or, high densities limit settling possibilities of immigrants. Dispersal is indeed negatively density dependent in tsetse flies (De Meeûs et al., 2019).

In Bonon, the increase of genetic variance after control campaigns suggests that flies sampled after controls may come from surrounding sites, at least partly, as was observed 35 years ago for the same species in the same area (Randolph et al., 1984; Rogers and Randolph, 1984; Rogers et al., 1984). The drastic drop of >91% in flies' density may have freed up space for surrounding flies. So the area that seemed saturated before control can receive new colonizers. Because of recent treatment and/or immigration, T1–4 subsamples were harvested from subpopulations in strong disequilibrium where effective population density and isolation by distance are hard to measure. The very odd results obtained for *D*_*e*_T_ and *δ*_T_ after treatment can illustrate this. Alternatively, it may also reflect the inappropriateness of defining the surface occupied by a subpopulation with the threshold distance for genetic subdivision. The relevance of such method was indeed strongly questioned (Rousset, 1997). On the other hand, before control campaigns, these *D*_*e*_T_ and *δ*_T_ appeared very close to values using census population sizes. When we used the sampling surface, effective population densities appeared significantly smaller than census density before control but dropped to values similar to those computed with census population sizes, while dispersal remained unchanged but with an important increase of the confidence interval. This may reflect a classical result where *N*_*e*_ < *N*_*c*_ (e.g. (Krafsur and Maudlin, 2018)), before treatment, while both *N*_*e*_ and *N*_*c*_ drop to similar very low values after control and only the variance of dispersal is affected but not the average. So, estimating effective population density and dispersal with the sampling surface method may be much more accurate than the threshold distance for genetic subdivision method.

Locus GpCAG seems selected by the control technique used in Bonon, by a mechanism that may include either insecticide resistance, behavioral avoidance of trapping device or another unknown mechanism. This would mean that some flies with specific GpCAG profiles (allele 219) were able to escape from control measures: either because this trinucleotide locus may code for something, or because it is part of a selected gene as an intronic sequence, or very close to it. It would be interesting to identify what caused this result. We have been unable to find to what corresponded the sequence where this locus is exactly. Indeed, a “highly similar sequences (megablast)” in GenBank at https://blast.ncbi.nlm.nih.gov/Blast.cgi (with the accession number AY033512.1) outputted no result but the sequence deposited by Baker and Krafsur (2001). A discontiguous megablast in GenBank, using the Program BLASTN 2.8.1+ (Zhang et al., 2000) did not provide very useful information. It matched at 75% with a nuclear receptor coactivator of *Salvelinus alpinus* (a salmonid fish), with a putative mediator of RNA polymerase II transcription subunit 26 of *Ceratitis capitata* (at 69%) and *Rhagoletis zephyria* (at 72%), two Dipteran insects of the sub-order Brachycera like tsetse flies. Deeper investigations would be required to decipher the mechanism involved in the possible resistance associated with GPCAG allele 219. Insecticide resistance has never been reported in tsetse flies to our knowledge and we could find nothing more than recommendations on that issue in an old FAO report (Georghiou et al., 1993) and assumptions that the likelihood of insecticide resistance evolution is negligible in tsetse (Krafsur and Maudlin, 2018). Moreover, the slight superiority of subdivision measured at GPCAG locus even before the control campaign presented here suggests the signature of preceding past treatments. This can come from the different sampling campaigns that have preceded this study between 2000 and 2001 (Courtin et al., 2005; Ravel et al., 2007), using Vavoua traps without insecticide, which resulted in drastic reduction in tsetse densities (Courtin et al., 2005). Alternatively, the massive use of insecticides and pesticides in cocoa and coffee plantation may also explain these results, hypothetically. Resistance evidenced here is possibly behavioral. Insecticide-avoidance behavior is known for different mosquito species (Chareonviriyaphap et al., 1997; Chareonviriyaphap et al., 2013; Tainchum et al., 2013; Porciani et al., 2017). As for tsetse flies, physiological or behavioral resistance is only suspected, if not speculated (Georghiou et al., 1993) but, to our knowledge, was never documented.

If resistance is behavioral, the fact that a substantial proportion of resistant tsetse flies harboring the GpCAG-219 allele were captured in the trapping device after treatment suggests: i) that resistance is not absolute but statistical; and ii) that the proportion of resistant tsetse flies must be much higher in the pool of uncaptured flies.

More researches are needed to locate this locus in the *Glossina* genomes and check if it is in or near a coding gene, determine its nature, and clarify if this trinucleotide microsatellite itself is responsible for what we have evidenced in the present paper.

## 5 Conclusion

The work presented here shows that control campaigns has modified tsetse flies population structure. Although it has allowed reducing considerably tsetse fly's densities, it may also have selected for the emergence of flies resisting the treatment by a mechanism that remains to be identified. This result should be taken into account and new strategies developed to prevent reinvasion.

The following are the supplementary data related to this article.

Supplementary Table S1 Raw data of *Glossina palpalis palpalis* of Bonon and Sinfra HAT foci. The different columns correspond to (in order): year, month and day of sampling; sampling site, treatment (To-T4), cohort (generations 1-11), trap names, latitude and longitude (in decimal degrees), individual labels, sex, the combination of treatment-Site-Trap to define subsamples and the different genotypes at the 10 loci (two columns per locus). The first row contains column headers and the other rows correspond to the different tsetse fly individuals. Supplementary Table S1

Supplementary Table S2 Microsatellite markers used for genotype Glossina palpalis palpalis (T anneal: annealing temperature in °C; nb of cycles: number of cycles). Supplementary Table S2
